# Coordination Complex Transformation-Assisted Fabrication for Hollow Chestnut-Like Hierarchical ZnS with Enhanced Photocatalytic Hydrogen Evolution

**DOI:** 10.3390/nano9020273

**Published:** 2019-02-15

**Authors:** Leilei Xu, Yuwei Ao, Bin Guan, Yun Xiang, Jianguo Guan

**Affiliations:** State Key Laboratory of Advanced Technology for Materials Synthesis and Processing, International School of Materials Science and Engineering, Wuhan University of Technology, 122 Luoshi Road, Wuhan 430070, China; xull@whut.edu.cn (L.X.); ayw808@126.com (Y.A.); 245627@whut.edu.cn (B.G.); xiangyun0608@163.com (Y.X.)

**Keywords:** hierarchical structure, wurtzite ZnS, coordination complex transformation-assisted growth, photocatalytic hydrogen production

## Abstract

Hierarchical nanostructures (HNs) are possibly endowed with novel properties due to their complex three-dimensional (3D) structures. Here, we provide a novel stepwise growth strategy of Coordination Complex Transformation-Assisted Growth for fabricating HNs. By using this, we prepare a new wurtzite ZnS HNs-hollow chestnut-like hierarchical microspheres (HCHMs), which are mesoporous hollow microspheres with single crystalline nanorods arrayed densely and radially from the centre. The HCHMs formation depends on the stepwise decomposition of the two Zn^2+^ complexes ([Zn(en)_m_(H_2_O)_2(3−m)_]^2+^ and [Zn(en)_m_(NH_3_)_2(3−m)_]^2+^, natural number m < 3). As the reaction proceeds, [Zn^2+^] has been distinctly reduced due to the transformation from [Zn(en)_m_(H_2_O)_2(3−m)_]^2+^ to [Zn(en)_m_(NH_3_)_2(3−m)_]^2+^ with a high stability constant, leading to a low crystal growth rate to obtain single crystalline nanorods. Additionally, the generated bubbles (CO_2_, NH_3_) acting as a template can induce the generation of hollow structure. The as-prepared ZnS HCHMs show an enhanced photocatalytic hydrogen evolution activity due to the single crystalline wurtzite phase and the high surface area contributed by the hollow hierarchical structures, as well as the mesoporosity. The versatility of the coordination complex transformation-assisted growth strategy will open up new possibilities for fabricating HNs, especially for those transition metal ions with excellent complex capabilities.

## 1. Introduction

3D HNs constructed from building blocks of nanoparticles, nanorods, nanoplates, nanoribbons or nanobelts generally possess the low density, high crystallinity and large specific surface area, as well as the unique optical, electric and magnetic properties [1,2,3,4]. Thus, they have great potentials in microelectronics, photocatalysis, biosensors and information storage and have attracted much attention of chemists and material scientists [5,6,7,8,9]. Compared with the widely used chemical vapor deposition approach, chemical solution phase synthesis shows distinct advantages, such as low-cost and large-scale production, which is promising to attain 3D HNs including semiconductors, metals, alloys and so forth [10,11,12,13,14].

ZnS, as an important semiconductor, has promising potentials in optoelectronic and luminescent devices due to its polar surface, wide direct bandgap, high transport ability, excellent thermal stability, electronic mobility and so forth [15,16,17]. Hexagonal wurtzite ZnS (WZ) is thermodynamically metastable phase and has an inner-polar electric field to facilitate the separation of photogenerated electrons and holes, and thus accelerate their transition to the surfaces. As a result, the wurtzite phase should have a better theoretical photocatalytic activity than the sphalerite phase in ZnS [18,19,20]. In the last two decades, many efforts have been made to advance its performances by regulating the morphologies and nanostructures including one dimensional (1D) nanotubes, nanowires and nanorods [21,22,23,24], as well as two dimensional (2D) nanosheets, nanobelts [25,26], porous nanosheets [27,28,29,30] and so forth. However, the remarkable intrinsic anisotropy of WZ to some extent restricts the synthesis of 3D HNs, because it usually favours the c-axis growth in a relatively slow growth rate to form 1D or 2D nanostructures or rapidly generates 0D particles under a high [Zn^2+^] [31,32,33]. Subsequently, a few WZ HN microspheres have been reported, which were also completely self-assembled from 1D nanorods or 2D nanosheets [20,34,35]. Although hollow ZnS urchin-like HNs with core-shell feature were obtained later, the synthesis route involving heating Zn powder under argon atmosphere, surface sulfuration and vaporization of Zn cores was complicated and the synthesis conditions were relatively harsh, such as high temperature sulfuration [36]. 

In this article, we reported a coordination complex transformation-assisted growth combining with a gas-bubble induced strategy for fabricating WZ HNs composed of mesoporous hollow microsphere cores and dense single crystalline nanorod shells. In our protocol, the coordination complex transformation from the complex of [Zn(en)m(H_2_O)_2(3−m)_]^2+^ to the more stable [Zn(en)_m_(NH_3_)_2(3−m)_]^2+^ has been carried out to regulate the [Zn^2+^] in the solution, which is significant for the growth kinetic of ZnS HCHMs. Therefore, the stepwise growth of WZ has been obtained to form chestnut-like hierarchical structures. The generated gas-bubbles during the preparation can be acted as the template for the formation of hollow structure. The as-obtained ZnS HCHMs with single crystalline, mesoporous and hierarchical characters show a remarkably structure-induced improvement of photocatalytic H_2_ production. This stepwise complexation strategy is significantly different from the widely used assembly, the surfactant-mediated growth or the precursor morphology replication and provides an illuminating insight into the synthesis of hierarchical structures. Additionally, the as-prepared ZnS HCHMs will be of great interest for applications in catalysis, adsorption, optoelectronics, nanotechnology and so on.

## 2. Materials and Methods 

### 2.1. Materials

Thiourea (CS(NH_2_)_2_, 99%) was bought from Shanghai Lingfeng Chemical Reagent Co., Ltd. (Shanghai, China) Zinc acetate dihydrate (Zn(Ac)_2_·2H_2_O, 99%), anhydrous ethylenediamine (en, 99%), sodium sulphite (Na_2_SO_3_, 97%) and anhydrous ethanol (EtOH, 99.7%) were purchased from Sinopharm Chemical Reagent Co., Ltd. Sodium sulphide (Na_2_S·9H_2_O, 98%) was got from Shanghai Tongya Chemical Technology Development Co., Ltd. (Shanghai, China) Deionized water was used in all reactions.

### 2.2. Sample Preparation

In a typical synthesis of ZnS HCHMs, 0.06 mmol Zn(Ac)_2_·2H_2_O and 3 mmol CS(NH_2_)_2_ were dissolved into 12 mL en aqueous solution with a volume ratio of en to water (*V*_en_/*V*_water_) of 1:2. The mixed solution was transferred into a 15 mL Teflon-lined autoclave and then heated at 200 °C for 12 h. After the autoclave was took out and cooled to the room temperature in air, the obtained products was subsequently rinsed with deionized water and EtOH to remove the adsorbed solvent and reactants. Finally, the obtained powders were dried at 60 °C for 6 h under vacuum conditions.

### 2.3. Sample Characterization

The morphologies of the as-prepared samples were characterized by field emission scanning electron microscope (FE-SEM; S-4800, Hitachi, Ltd., Tokyo, Japan). The acceleration voltage is 10.0 kV. The crystalline structure characterization of the as-prepared samples were recorded on X-ray diffraction (XRD; D/MAX-RB, Rigaku Co., Tokyo, Japan), using Cu Kα radiation (λ = 1.5418 Å). The test range was from 10° to 80°. The JEM-2100F (JEOL Ltd., Tokyo, Japan) was used to obtain transmission electron microscopy (TEM) and high resolution transmission electron microscopy (HRTEM) images as well as the corresponding selected area electron diffraction (SAED) patterns. The accelerating voltage is 200 kV. The as-prepared samples after being dispersed in ethanol for 30 min were dropped on a carbon film coating on a Cu grid. The FTIR spectrometer (Nicolet-60SXB, Thermo Nicolet Co., Madison, WI, USA) was used to obtain the Fourier transform IR (FTIR) spectrum. The range is from 400 to 4000 cm^−1^ and the resolution is 4 cm^−1^. The N_2_ adsorption-desorption isotherm of the as-prepared samples was obtained by using a Micromeritics ASAP 2020 nitrogen adsorption instrument (Micromeritics Instrument Co., Norcross, GR, USA). The pore size distribution was analysed by using the desorption isotherm according to the Barret-Joyner-Halender (BJH) method. The pore structure was assumed as a cylindrical pore model. The as-prepared sample was tested after degassing at 200 °C for 12 h. The light absorption properties of the as-prepared samples were obtained on a UV-Vis spectrometer (UV-2550, Shimadzu Co., Kyoto, Japan) using BaSO_4_ as a reflectance standard. 

### 2.4. Photocatalytic Activity

In a typical photocatalytic test, the as-prepared samples (50 mg) were dispersed in a Na_2_S (0.35 M) and Na_2_SO_3_ (0.25 M) mixed aqueous solution (30 mL) under a constant stirring. Prior to irradiation, the closed gas-circulation system and the suspension in the reaction cell were purged for 30 min in order to remove most of the dissolved oxygen in the solution. Subsequently, a 300 W xenon lamp (PLS-SXE300, Beijing Trusttech Co. Ltd., Beijing, China) with an IR filter was placed on an external-irradiation Pyrexcell of the circulation system about 20 cm. The average light intensity is 5 mW·cm^−2^ and the wavelength range is from 320 to 780 nm. The emission spectrum can be found in Figure S1 in the Supplementary Materials. The diameter of the Pyrexcell and the facula size of the xenon lamp are both almost 63 mm. The evolved H_2_ was in situ recorded by using a GC 7890-II gas chromatograph (TECHCOMP Ltd., Shanghai, China), which was connected to the closed gas-circulation system. The detector is thermal conductivity detector (TCD, TECHCOMP Ltd., Shanghai, China), the chromatograph column is MS-5A and the carrier is nitrogen.

## 3. Results and Discussion

**Figure 1:**
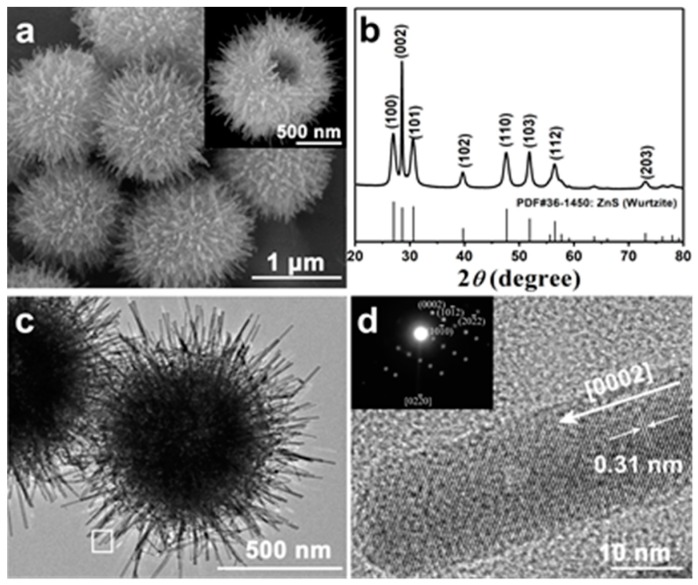
(**a**) SEM images, (**b**) XRD pattern and (**c**) TEM image of the as-prepared ZnS HCHMs; (**d**) HRTEM image and SAED pattern of a single ZnS nanorod taken from (**c**) enclosed by a square (inset).

Figure 1a demonstrates that the as-obtained products have a uniform chestnut-like hierarchical structure being composed of nanothorns with 10–30 nm in diameters and 200–250 nm in lengths. The nanothorns are radially arrayed from the centre of microspheres. The size of the hierarchical microspheres is about 1 μm. The inset image of a broken microsphere shows a distinct hollow structure and the mesoporosity of the inner shell layer. The XRD pattern of the ZnS HCHMs shown in Figure 1b is indexed to wurtzite phase (JCPDS no. 36-1450) without any impurities. The obvious enhanced peak intensity of (002) in the as-prepared sample pattern indicates an orientation growth along [0002] of nanothorns. According to the Debye Scherrer formula, the average grain size of the as-prepared ZnS is calculated to be about 45 nm by the full width at half maximum (FWHM) of the (002) diffraction peak [37]. As demonstrated in Figure 1c, the as-prepared ZnS HCHMs are composed of a porous spherical core and high-density nanorods arraying on the surface of microspheres. Due to the small size of hollow parts, there is no obvious manifestation except the little reduction of the contrast in the core. The single nanorod shown in the HRTEM image (Figure 1d) is from Figure 1c enclosed by a square. The interplanar spacing of 0.31 nm corresponds to the (0002) crystal plane of WZ, which is consistent with the SAED pattern shown in the inset of Figure 1d, indicating the growth direction [0002] of the nanorod units. Moreover, the SAED pattern also indicates its obvious single-crystalline feature. The above characterizations obviously demonstrate that a WZ HNs composed of hollow mesoporous cores and single-crystalline nanorod shells have been synthesized.

**Figure 2:**
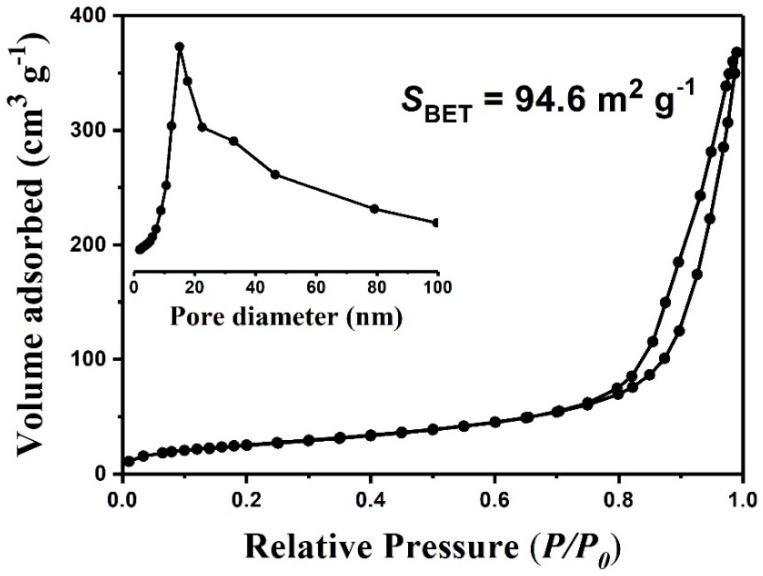
The N_2_ adsorption–desorption isotherm of the as-prepared ZnS HCHMs and the inset is the pore size distribution curve with the corresponding *S*_BET_.

The as-prepared ZnS HCHMs exhibits a typical type IV N_2_ adsorption-desorption isotherm combining with a H3 hysteresis loops, indicating its obvious mesoporosity (Figure 2). The corresponding pore-size distribution curve shown in the inset of Figure 2 clearly indicates a wide pore-size range of the as-prepared ZnS HCHMs. The smaller pore size of ~20 nm may derive from the aggregation of ZnS crystalline grains in the porous spherical core and the larger pores may be due to the hollow feature. The BET surface area (*S*_BET_) of the ZnS HCHMs (94.6 m^2^·g^−1^) observed in Figure 2 is much larger than that of ZnS microspheres (49 m^2^·g^−1^) completely self-assembled from 1D nanorods [34]. This may provide more active sites for photocatalytic reaction and thus enhance the photocatalytic activity for H_2_ production.

**Figure 3:**
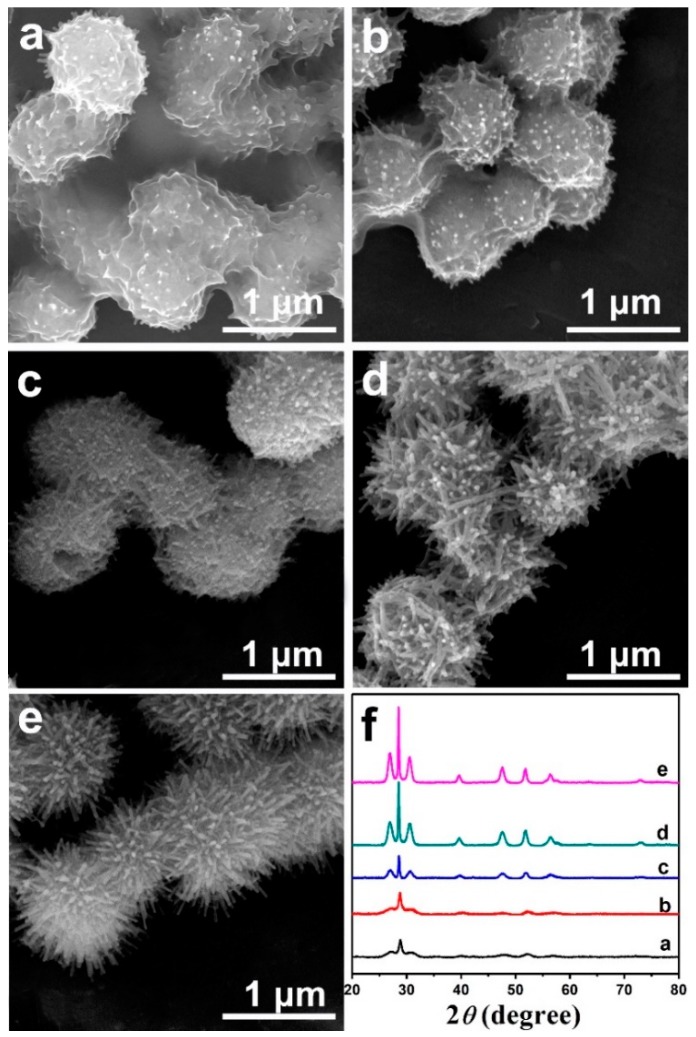
SEM images (**a**–**e**) and XRD patterns (**f**) of the samples obtained at 200 °C with a *V*_en_/*V*_water_ of 1:2 for (**a**) 20 min, (**b**) 30 min, (**c**) 2 h, (**d**) 6 h and (**e**) 24 h, respectively.

In order to clear the formation of the as-prepared ZnS HCHMs, the time (*t*)-dependent morphologies and crystalline phases of the samples are shown in Figure 3. At *t* = 20 min, a high [Zn^2+^] in the solution results in the aggregation of ZnS crystalline grains to form nearly spherical structures. The spherical particles possess a rough surface with a diameter of about 650–700 nm (Figure 3a). With increasing *t* to 30 min, the size of particles increases to 700–800 nm and simultaneously some tiny nanorods occur on the surface of the spherical particles (Figure 3b). At *t* = 2 h, the surface nanorods grow and become compact. Meanwhile, an obvious hollow structure can be observed from a broken particle (Figure 3c). Increasing *t* to 6 h, it can be observed that the nanorods grow radially from the spherical core and the chestnut-like microspheres are obtained with a diameter of ca. 900 nm. The diameters and the lengths of the nanorods are about 20 nm and 150 nm, respectively. Further increasing *t* to 24 h, the sample shows a distinct HCHM structure without any changes compared with the typical sample. From Figure 3f, we can observe that the samples obtained at different *t* are matched well with the wurtzite phase of ZnS and the crystallinities increase gradually with increasing *t*. 

According to the time-dependent contrastive experiments, the formation of the as-prepared ZnS HCHMs is mainly composed of two processes involving the aggregation of particles to generate the spherical core and the subsequent quasi-equilibrium growth of nanorod shells. At the primary step of the solvothermal reaction, Zn^2+^ ions in the solution first react to form [Zn(en)_m_(H_2_O)_2(3−m)_]^2+^ complex ions (natural number m < 3) in the existence of H_2_O (Equation (1)) [38].

Zn^2+^ + m en + 2(3 − m)H_2_O → [Zn(en)_m_(H_2_O)_2(3−m)_]^2+^, (1)

With the increase of temperature, the reaction of thiourea with water produces S^2−^ and bubbles CO_2_ and NH_3_ following Equation (2) [39], while complex [Zn(en)_m_(H_2_O)_2(3−m)_]^2+^ begins to dissolve in the solution because of its low stability constant (Equation (3)), resulting in the existence of Zn^2+^ ions at a relatively high concentration. This allows vast heterogeneous nucleation and aggregation of ZnS nanoparticles in the bubble-liquid interface (Equation (4)). Consequently, hollow spherical particles are generated.

SC(NH_2_)_2_ + 2H_2_O → S^2−^ + 2H^+^ + CO_2_↑ + 2NH_3_↑, (2)

[Zn(en)_m_(H_2_O)_2(3−m)_]^2+^ ⇌ Zn^2+^(high concentration) + m en + 2(3 − m)H_2_O, (3)

Zn^2+^(high concentration) + S^2−^ → ZnS (nanoparticles), (4)

With the above reactions (Equations (2)–(4)) proceeding continuously, large number of the generated NH_3_ are dissolved in the aqueous solution (Equation (5)). This makes complex [Zn(en)_m_(H_2_O)_2(3−m)_]^2+^ with low stability constant transform into highly stable ternary complex [Zn(en)_m_(NH_3_)_2(3−m)_]^2+^ ions with prolonging *t* according to Equation (6) [40]. Thus, the Zn^2+^ concentration ([Zn^2+^]) is significantly lowered because of the large stability constant of [Zn(en)_m_(NH_3_)_2(3−m)_]^2+^ ions (Equation (7)). The decreased [Zn^2+^] in the solution can induce the quasi-equilibrium growth of nanorods located on the surface of the hollow spherical particles. As a result, ZnS single crystalline nanorods are grown radially.

NH_3_ + H_2_O → NH_3_ (aq), (5)

[Zn(en)_m_(H_2_O)_2(3−m)_]^2+^ + 2(3 − m)NH_3_ (aq) ⇌ [Zn(en)_m_(NH_3_)_2(3−m)_]^2+^ + 2(3 − m)H_2_O, (6)

[Zn(en)_m_(NH_3_)_2(3−m)_]^2+^ ⇌ Zn^2+^(low concentration) + m en + 2(3 − m) NH_3_, (7)

Zn^2+^(low concentration) + S^2−^ → ZnS (nanorods), (8)

Therefore, the formation mechanism of the ZnS HCHMs can be depicted as follows. At the primary step of the reaction, the decomposition of [Zn(en)_m_(H_2_O)_2(3−m)_]^2+^ with a low stability constant generates a large amount of Zn^2+^ ions (Zn^2+^(I)) in the solution. Because of the high [Zn^2+^(I)], ZnS clusters primarily aggregate to form spherical cores. Meanwhile, the gas-bubbles from the decomposition of thiourea induce the formation of hollow structure [41,42]. Following with the generation of [Zn(en)_m_(NH_3_)_2(3−m)_]^2+^ and the formation of the spherical cores, [Zn^2+^] in the solution has been greatly decreased to induce the quasi-equilibrium growth of nanorods. Under this quasi-equilibrium state, small ZnS dendrites grow from the protuberances on the rough surface of the spherical cores according to a tip-growth mechanism [1,43]. The anisotropic growth of ZnS nanorods along the [0002] direction selectively occur at these high-energy protuberances. As a result, the as-prepared ZnS HCHMs are finally formed after 12 h for the growth of ZnS nanorods. 

**Figure 4:**
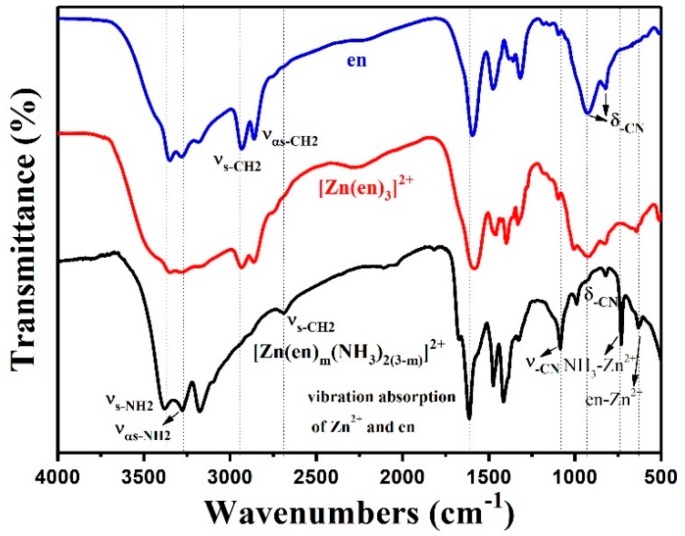
FTIR spectra of pure en, [Zn(en)_3_]^2+^ and the dried product obtained from the supernatant liquid after the typical solvothermal reaction for 20 min.

To further confirm the evolution of ZnS HCHMs, the supernatant liquid of the solvothermal reaction after 20 min was characterized by FTIR. The FTIR spectra of pure en and [Zn(en)_3_]^2+^ were also given in Figure 4. The peaks at 3379 cm^−1^ and 3277 cm^−1^ match with the ν_s-NH2_ and ν_αs-NH2_, respectively and the peak at 3176 cm^−1^ is caused by hydrogen-bonding association of –NH_2_ [44]. Comparing with en and the binary complex ([Zn(en)_3_]^2+^), the three peaks with a clear vibration strengthening indicates the possible formation of [Zn(en)_m_(NH_3_)_2(3−m)_]^2+^ after 20 min. The ν_s-CH2_ and ν_αs-CH2_ appearing at 2933 cm^−1^ and 2861 cm^−1^ in the parent ligand of en are shifted towards the lower frequency at 2689 cm^−1^ in the [Zn(en)_m_(NH_3_)_2(3−m)_]^2+^. Moreover, there is an additional peak at 1672 cm^−1^ in the product, which can be ascribed to the frame composed of en and Zn^2+^ vibration absorption. The peaks at 1612 cm^−1^, 1474 cm^−1^ and 822 cm^−1^ are respectively assigned to δ_-NH2_, δ_-CH2_ and δ_-CN_, while the three peaks between 1420–1000 cm^−1^ match with ν_-CN_. The obvious strengthen and shift towards the lower frequencies of the three peaks indicate a higher chelating nature of [Zn(en)_m_(NH_3_)_2(3−m)_]^2+^. Additionally, the peak at 731 cm^−1^ and 632 cm^−1^ can be assigned to NH_3_ → Zn^2+^ and en → Zn^2+^ stretching bands in the ternary complex. All of these clearly confirm the generation of [Zn(en)_m_(NH_3_)_2(3−m)_]^2+^ in the solvothermal process.

**Figure 5:**
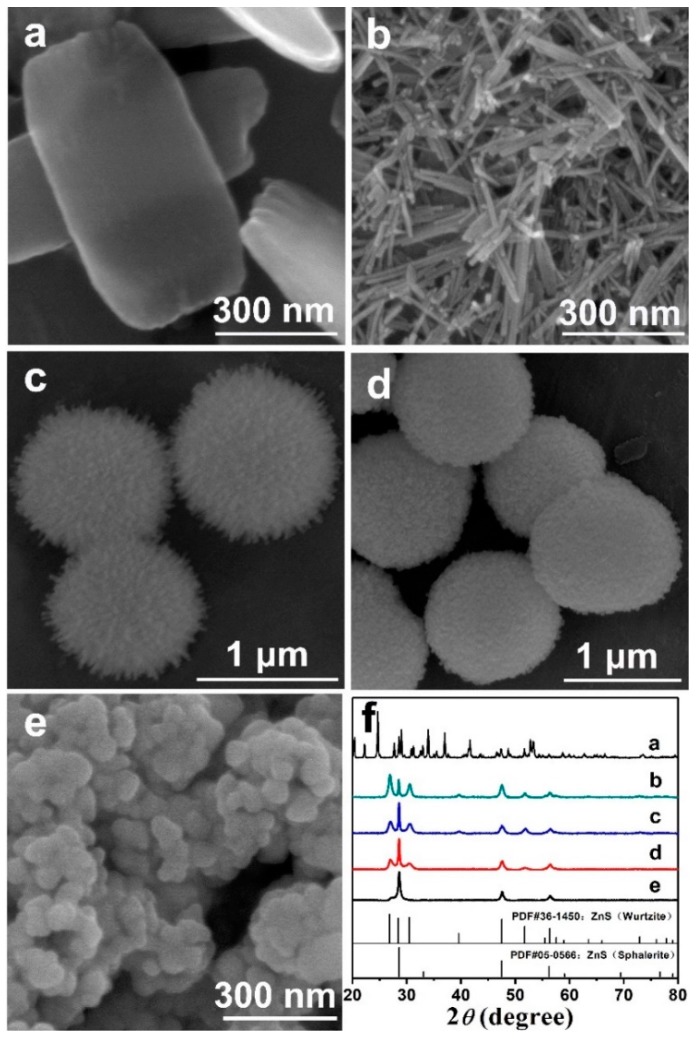
SEM images (**a**–**e**) and XRD patterns (**f**) of the samples obtained at 200 °C for 12 h with different *V*_en_/*V*_water_: (**a**) pure en, (**b**) 1:1, (**c**) 1:3, (**d**) 1:5, (**e**) pure water.

**Scheme 1:**
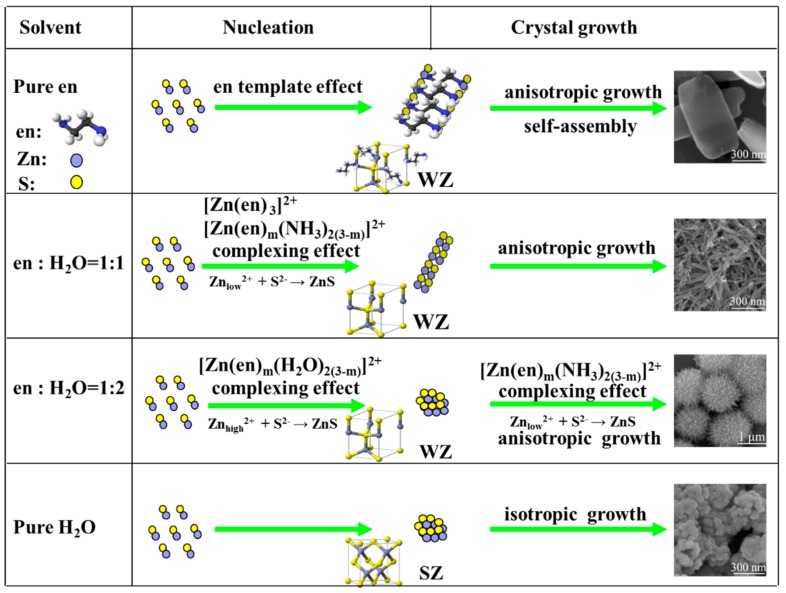
The schematic illustration of structural control processes of ZnS micro/nanostructures.

In view of the proposed formation mechanism, regulating reaction kinetics is the key for the obtained HCHMs which is closely related to the amounts of en and S sources. Therefore, the effects of *V*_en_/*V*_water_ as well as the concentration of thiourea on compositions and structures are evaluated, respectively. Figure 5 shows SEM images and XRD patterns of the as-prepared samples obtained at different *V*_en_/*V*_water_. It can be observed that in pure en only ZnS(en)_0.5_ nanosheets are generated (Figure 5a,f) because of the intercalation characters of en [18,19,20,21,29]. When *V*_en_/*V*_water_ is 1:1, ZnS nanorods of 10–20 nm in diameters and 150–200 nm in lengths are produced (Figure 5b,f). Typical ZnS HCHMs are only obtained when the volume ratio reaches 1:2. Further decreasing the content of en, the length of nanorods becomes smaller (Figure 5c,d). If the solvent is pure H_2_O, nearly spherical sphalerite ZnS aggregated nanoparticles are got (Figure 5e,f). The structure control processes of ZnS are proposed in Scheme 1.

In the absence of H_2_O (Figure 5a), Zn^2+^ ions generated from slow decomposition of [Zn(en)_3_]^2+^ react with S^2−^ to form ZnS(en)_0.5_. The temple effect of en induce the generation of ZnS(en)_0.5_ nanosheets via an oriented growth [39]. Introducing H_2_O, the [Zn(en)_m_(H_2_O)_2(3−m)_]^2+^ forms firstly (Equation (1)) and then transforms into relatively stable [Zn(en)_m_(NH_3_)_2(3−m)_]^2+^ complex (Equation (6)). However, when we cut down the concentration of en contrarily (*V*_en_/*V*_water_ = 1:3 and/or 1:5), the [Zn(en)_m_(H_2_O)_2(3−m)_]^2+^ instead of [Zn(en)_m_(NH_3_)_2(3−m)_]^2+^ becomes the main complex during the reaction. As a result, the length of the thorns in the HMCMs becomes shorter and shorter. While in pure water without producing any complexes (Figure 5e), the high concentration of Zn^2+^ leads to a rapid nucleation and crystal growth rate and thus gives rise to the isotropic growth to obtain agglomerated sphalerite ZnS nanoparticles due to its relatively lower surface energy. Therefore, the *V*_en_/*V*_water_ in the solution greatly impacts the generation of [Zn(en)_m_(H_2_O)_2(3−m)_]^2+^ and [Zn(en)_m_(NH_3_)_2(3−m)_]^2+^ in the solvothermal process and thus regulates morphology and structure of the samples. This can be further evidenced by introducing excessive NH_3_∙H_2_O in the synthesis of ZnS HCHMs, in which the anisotropic nanorod structure forms instead of hierarchical structure (Figure S2 in the Supplementary Materials). The morphology of the as-prepared sample is closely related to the relative amounts of the two complex, which further depends on the dissolved amount of NH_3_ in the solution according to Equation (6).

**Figure 6:**
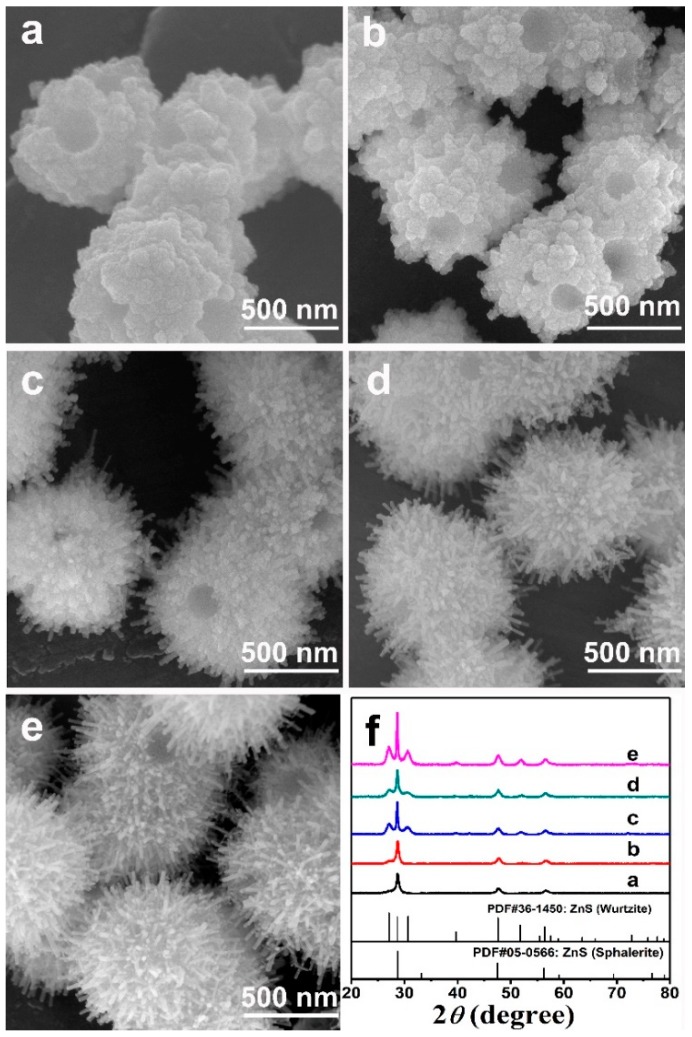
SEM images (**a**–**e**) and XRD patterns (**f**) of the samples obtained at 200 °C for 12 h with *V*_en_/*V*_water_ = 1:2 under different molar ratio of Zn to S (*M*_Zn_/*M*_S_): (**a**) 1:3, (**b**) 1:5, (**c**) 1:10, (**d**) 1:15 and (**e**) 1:20.

When molar ratio of Zn to S (*M*_Zn_/*M*_S_) in the solvothermal process is 1:3 or 1:5, a decreased content of thiourea reduces the concentration of NH_3_, which remarkably influence the generation of [Zn(en)_m_(NH_3_)_2(3−m)_]^2+^. Therefore, the Zn^2+^ ions in the solution mainly derive from the decomposition of [Zn(en)_m_(H_2_O)_2(3−m)_]^2+^. As a consequence, sphalerite aggregated microparticles (Figure 6a,b,f) are obtained. Further decreasing *M*_Zn_/*M*_S_ from 1:10 to 1:50, the expected HMCMs can be prepared and the surficial nanorods become denser and longer (Figure 6c–e). The results can further validate our speculation of the formation mechanism of ZnS HCHMs.

**Figure 7:**
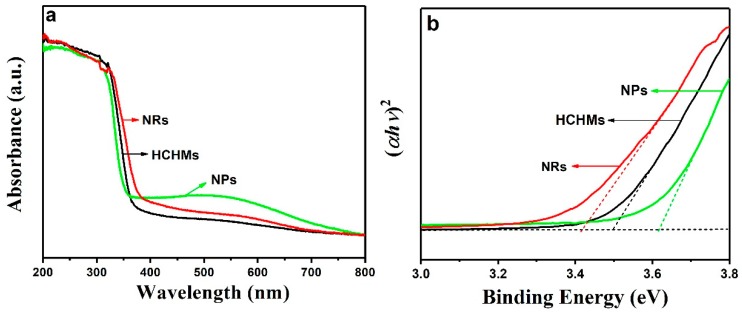
(**a**) UV-Vis DR spectra and (**b**) the corresponding plot of (*αhν*)^2^ versus binding energy of the as-prepared ZnS HCHMs as well as ZnS nanorods (NRs) obtained at *V*_en_/*V*_water_ = 1:1 and ZnS nanoparticles (NPs) obtained in pure water.

Figure 7a illustrates the UV-Vis DR spectrum of the ZnS HMCMs. The ZnS nanorods (NRs) obtained at *V*_en_/*V*_water_ = 1:1 and the sphalerite ZnS nanoparticles (NPs) obtained in pure H_2_O were also tested for comparison. The absorption of the three samples at 370 nm can be ascribed to the intrinsic absorption of ZnS. The absorption centred at about 550 nm in the visible-light region derives from zinc vacancies [31]. The strong absorption of ZnS NPs indicates that it has more zinc vacancies than other two samples that can be explained by its fast reaction kinetic in pure water. By using the transformed Kubelka-Munk function, the corresponding bandgap energies of the as-prepared three samples are calculated. As presented in Figure 7b, the bandgap of sphalerite ZnS NPs is 3.62 eV, while the wurtzite ZnS NRs and HCHMs have bandgap energies of 3.42 and 3.50 eV, respectively. This indicates that the wurtzite ZnS NRs and HCHMs possess the wider light absorption than the sphalerite ZnS NPs. Additionally, the slight band edge blue shift of the as-prepared ZnS HCHMs compared with the NRs may be due to the smaller size of the nanothorns.

**Figure 8:**
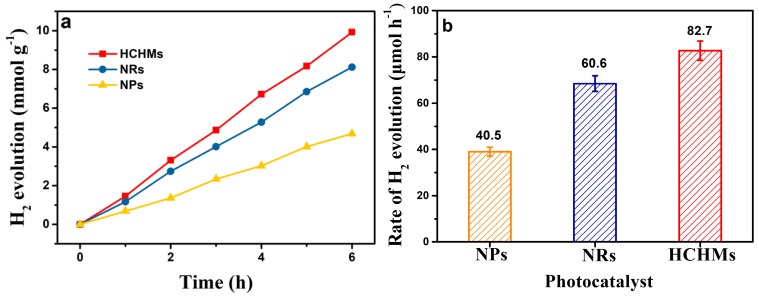

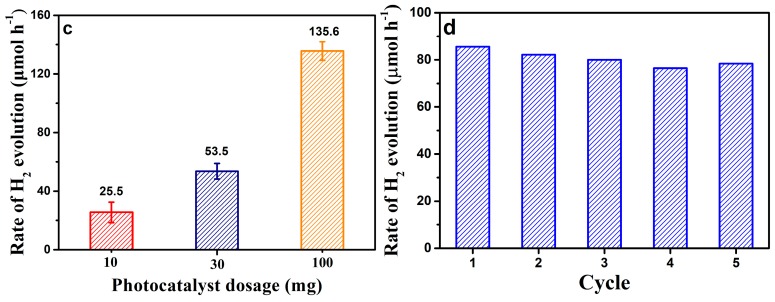
(**a**) The amount and (**b**) the rate of H_2_ production of the as-prepared ZnS HCHMs and the ZnS NRs as well as the sphalerite ZnS NPs under UV light irradiation with 50 mg photocatalyst dosage; (**c**) The effect of photocatalyst dosages on the rate of H_2_ production and (**d**) Photocatalytic stability test of the as-prepared ZnS HCHMs. Reaction solution: 0.35 M Na_2_S and 0.25 M Na_2_SO_3_ aqueous solution; Light source: 300 W xenon lamp.

Figure 8a,b shows the photocatalytic H_2_ production activities of the as-prepared ZnS HCHMs and the ZnS NRs as well as the sphalerite ZnS NPs. Contrast experiments show that no appreciable hydrogen production was detected without photocatalyst, further suggesting that hydrogen production under light irradiation is a photocatalytic reaction. The as-prepared ZnS HMCMs exhibits the highest photocatalytic activity. The maximum of H_2_-production rate is as high as 82.7 μmol·h^−1^ without the assistance of any co-catalysts, which is about 2 times and 1.5 times greater than that of sphalerite NPs and wurtzite NRs. The activity is also superior to the reported ZnS hierarchical structures as shown in Table S1 in the Supplementary Materials [20,45]. This enhanced photocatalytic activity for H_2_ evolution over ZnS HCHMs may be closely related to the composition and structure characteristics of the HMCM structure. The wurtzite crystal phase favours effective charge separation contributed by the inner-polar electric field [4]. The single-crystalline nature of nanorod units endow a rapid transfer of charge carriers and the porous structure and good dispersibility provide more active sites, rapid mass transfer and increased multiple light scattering [45,46]. 

The effect of photocatalyst dosages on photocatalytic H_2_ production is shown in Figure 8c. With increasing the photocatalyst dosage, the rate of H_2_-production increases correspondingly, while H_2_-production per unit mass photocatalyst decreases slightly. This decrease can be resulted from the inadequate utilization of active sites because of the limited reaction volume resulting in light shielding or bad dispersibility of photocatalysts. The stability test of the as-prepared ZnS HCHMs are evaluated by a long-term experiment for H_2_ evolution. The closed gas circulation system is vacuumed each 6 h during the whole test process and the rate of H_2_ evolution in each 6 h is evaluated depicted in Figure 8d, revealing that after each cycling no noticeable decrease in photocatalytic H_2_-production rate can be observed and about 90% H_2_ evolution can be obtained after being repeated for five cycles. 

## 4. Conclusions

In summary, a coordination complex transformation-assisted growth combining with a gas-bubble induced strategy has been developed to fabricate a novel hollow wurtzite ZnS chestnut-like hierarchical structure, which is composed of mesoporous hollow microsphere core and dense single crystalline nanorod shell. The formation of the hierarchical structure is mainly ascribed to the transformation from the complex ([Zn(en)_m_(H_2_O)_2(3−m)_]^2+^) to the more stable ([Zn(en)_m_(NH_3_)_2(3−m)_]^2+^) that reduces [Zn^2+^] in the solution as the reaction proceeded and thus leads to the stepwise growth of ZnS. Because of wurtzite crystal phase, the porous structure and single crystalline character, the as-prepared hierarchical structure exhibits an obvious structure-induced enhancement of photocatalytic activity for H_2_ production. This work not only provides an effective strategy to fabricate complex hierarchical nanostructures but also provides a novel ZnS HCHMs for exploring function applications. 

## Supplementary Materials

The following are available online at https://www.mdpi.com/2079-4991/9/2/273/s1, Figure S1: The emission spectrum (dark) of the Xe lamp employed in the hydrogen evolution measurement. Figure S2: The SEM image of ZnS obtained by adding NH_3_·H_2_O in the solvothermal process. Table S1: The comparison of the reported ZnS in surface physical-chemical properties and photocatalytic activities.
