# Supplementary material for: Coordination Complex Transformation-Assisted Fabrication for Hollow Chestnut-Like Hierarchical ZnS with Enhanced Photocatalytic Hydrogen Evolution
Source: Nanomaterials (Basel). 2019 Feb 15;9(2):273. doi: 10.3390/nano9020273 (PMC6409558; doi:10.3390/nano9020273)
Supplement: Supplementary file 1 [file nanomaterials-09-00273-s001.pdf]

Coordination complex transformation-assisted fabrication for hollow chestnut-like hierarchical ZnS with enhanced photocatalytic hydrogen evolution

Leilei Xu, Yuwei Ao, Bin Guan, Yun Xiang and Jianguo Guan*

State Key Laboratory of Advanced Technology for Materials Synthesis and Processing, International School of Materials Science and Engineering, Wuhan University of Technology, 122 Luoshi Road, Wuhan, 430070, P. R. China

* Correspondence: guanjg@whut.edu.cn; Tel.: +86-27-87218832

Supplementary Figure:

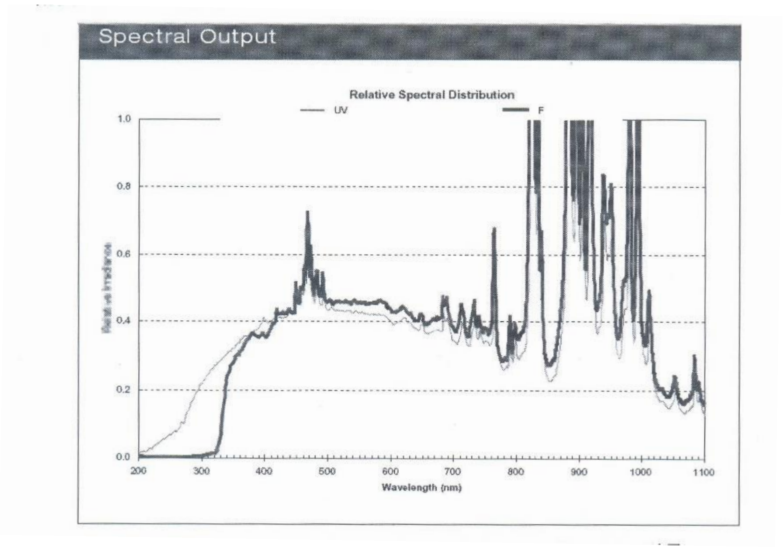

Figure S1. The emission spectrum (dark) of the Xe lamp employed in the hydrogen evolution measurement.

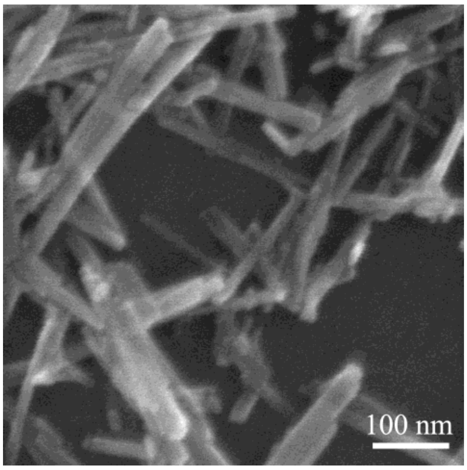

Figure S2. The SEM image of ZnS obtained by adding $NH_3{\cdot}H_2O$ in the solvothermal process.

Table S1. The comparison of the reported ZnS in surface physical-chemical properties and photocatalytic activities.

Sample	Synthesis	Phase Composition	S_{BET} (m^2 g^{-1})	Hydrogen evolution rate (μmol h^{-1} g^{-1})	Ref.
ZnS microspheres composed of interwoven nanosheets	Hydrothermal 230 °C 12 h	52 % wurtzite	100.2	1230, Four low-power UV-LED (3 W, 365 nm), 80.0 mW/cm^2	20
Urchin-like ZnS self-assembled nanorods	Hydrothermal 160 °C 24 h	wurtzite	49	-	34
Self-supported ZnS with interwoven nanosheets	Hydrothermal 230 °C 12 h	wurtzite	128	1602, 350 W Xe lamp, 20 mW/cm^2 .	43
Our sample	Hydrothermal 200 °C 12 h	wurtzite	94.6	1654, 300 W Xe lamp, 5 mW/cm^2	-
